# Seed Dispersal Anachronisms: Rethinking the Fruits Extinct Megafauna Ate

**DOI:** 10.1371/journal.pone.0001745

**Published:** 2008-03-05

**Authors:** Paulo R. Guimarães, Mauro Galetti, Pedro Jordano

**Affiliations:** 1 Departamento de Física da Matéria Condensada, Instituto de Física Gleb Wataghin, Universidade Estadual de Campinas, Campinas, São Paulo, Brazil; 2 Laboratório de Biologia da Conservação, Universidade Estadual Paulista (UNESP), Rio Claro, São Paulo, Brazil; 3 Integrative Ecology Group, Estación Biológica de Doñana, Consejo Superior de Investigaciones Científicas (CSIC), Sevilla, Spain; University of Zurich, Switzerland

## Abstract

**Background:**

Some neotropical, fleshy-fruited plants have fruits structurally similar to paleotropical fruits dispersed by megafauna (mammals >10^3^ kg), yet these dispersers were extinct in South America 10–15 Kyr BP. Anachronic dispersal systems are best explained by interactions with extinct animals and show impaired dispersal resulting in altered seed dispersal dynamics.

**Methodology/Principal Findings:**

We introduce an operational definition of megafaunal fruits and perform a comparative analysis of 103 Neotropical fruit species fitting this dispersal mode. We define two megafaunal fruit types based on previous analyses of elephant fruits: fruits 4–10 cm in diameter with up to five large seeds, and fruits >10 cm diameter with numerous small seeds. Megafaunal fruits are well represented in unrelated families such as Sapotaceae, Fabaceae, Solanaceae, Apocynaceae, Malvaceae, Caryocaraceae, and Arecaceae and combine an overbuilt design (large fruit mass and size) with either a single or few (<3 seeds) extremely large seeds or many small seeds (usually >100 seeds). Within-family and within-genus contrasts between megafaunal and non-megafaunal groups of species indicate a marked difference in fruit diameter and fruit mass but less so for individual seed mass, with a significant trend for megafaunal fruits to have larger seeds and seediness.

**Conclusions/Significance:**

Megafaunal fruits allow plants to circumvent the trade-off between seed size and dispersal by relying on frugivores able to disperse enormous seed loads over long-distances. Present-day seed dispersal by scatter-hoarding rodents, introduced livestock, runoff, flooding, gravity, and human-mediated dispersal allowed survival of megafauna-dependent fruit species after extinction of the major seed dispersers. Megafauna extinction had several potential consequences, such as a scale shift reducing the seed dispersal distances, increasingly clumped spatial patterns, reduced geographic ranges and limited genetic variation and increased among-population structuring. These effects could be extended to other plant species dispersed by large vertebrates in present-day, defaunated communities.

## Introduction

The strong evidence that positive density-dependent mortality occurs in seeds, seedlings, juvenile and adult plants in several different species suggests that seed dispersal is a key process in plant communities [1], [2]. Fruit traits certainly play a key role in the outcomes of interactions with seed dispersers, affecting the seed dispersal effectiveness (*sensu* [3]), and negative consequences for plant populations can be expected if the dispersal process is absent or impaired (e.g., [4], [5]). Yet, a large fraction of extant fleshy fruits show trait combinations that largely reflect their history of shared ancestry [6], not present-day adaptations to seed dispersers. In analogy with “ghosts of the competition past”, some combinations of fruit traits that can be found in extant communities suggest “ghosts of past mutualisms” [7], [8].

Many ecological studies have identified diverse interactions with the frugivorous fauna in different communities, usually ranging from a few to tens of species recorded feeding on the fruit of a given plant species [9], [10]. Even after recognizing that the plant-frugivore interaction can operate on exapted traits [11] of fruits, its outcomes have key functional effects on the demography, regeneration and gene flow patterns of the plants. Consequently, some structural patterns in fruits may be associated with distinct assemblages of seed dispersers [12]. In this context, the paradoxical existence of fruits with apparent adaptations for the dispersal by some groups of animals, in areas where these animals are now extinct, is an interesting topic with deep consequences for evolution, ecology and conservation of plant diversity. In fact, the loss of large mammals is still ongoing, and current defaunation scenarios have been shown to have serious consequences for plant populations [13]–[16].

Janzen and Martin [7] defined seed dispersal anachronisms as those dispersal syndromes with fruit traits and phenological patterns best explained by interactions with extinct animals and offered some striking examples of Neotropical fruits with anachronic traits (see also [8], [17]). These “unfit” species share fruit traits and phenological patterns that are at least in part not expected from their interactions with the extant frugivore community, but logically explained if we consider the extinction or local absence of the main frugivores. One of the seed dispersal anachronisms, the so-called megafaunal syndrome, includes fruits that were likely to be dispersed by now extinct large animals and has been the subject of considerable debate stemming from a lack of specific predictions and precise definitions [18]–[22]. There is a general consensus on the validity of the idea yet, “the ecological and evolutionary assumptions which prop up the megafaunal syndrome need rethinking” so that “an edifying refinement will evolve from the turmoil” [18, p. 860]. In this paper we revisit Janzen and Martin's [7] idea by analyzing the traits of megafaunal fruits in a comparative study. We expand this hypothesis with a rigorous characterization of the megafaunal syndrome and examine its ecological and life-history correlates. Rather than simply redefining it, we aim at identifying the levels at which the hypothesis can be supported, outlining the reasons that could explain plant persistence after loss of frugivores, and discussing the potential demographic and genetic consequences of the megafaunal syndrome.

Janzen and Martin [7] examined the hypothesis that frugivory by large extinct mammals like native horses, gomphotheres, ground sloths, and other Pleistocene megafauna offers an explanation to dispersal-related plant reproductive traits of Central American lowland forests. In their definition, key traits of megafaunal fruits include 1) overbuilt design, with large seeds protected mechanically by thick and hard endocarp and indehiscence, with nutrient-rich pulp and external similarity to fruits eaten by extant large African mammals; 2) phenological segregation of ripening times throughout the year; 3) fruits falling to the ground upon ripening; 4) fruits unattractive or not very attractive to arboreal or flying frugivores; 5) a large proportion of the fruit crop rots on the tree without being consumed; 6) frugivores include a large coterie of seed predators that might act sporadically as legitimate dispersers; 7) fallen fruits are avidly eaten by introduced horses, pigs, and cattle; and 8) natural habitats of the plant species today are alluvial bottoms on gentle slopes, usually along forest edges with grassland. The initial hypothesis of Janzen and Martin [7] was applied to Costa Rican vertebrate-dispersed species, but subsequent work has suggested that anachronic dispersal systems might occur worldwide [see e.g. 8], [22]–[Bibr pone.0001745-Herrera1] [24] and, specifically, megafaunal fruits can be found in different continents [12], [20], [25]–[29]. Janzen and Martin's idea [7] has been challenged with later analysis [18] and is implicitly assumed in the idea [24] that pterosaurs and pre-Pleistocene extinct megafauna [30], [31] had a central role in the dispersal of early angiosperm seeds. On the other hand, many of the species included in Janzen and Martin [7] have been reported to be dispersed by extant frugivores or abiotic vectors (e.g., water runoff) [32]. For example, while extremely limited dispersal can be observed in the field for a few species with megafaunal fruits (e.g., *Hymenaea courbaril)*, it is relatively frequent to record dispersal by gravity, water, scatter-hoarding rodents, monkeys and large-bodied birds or favored by human harvesting. It is important to note that we are not assuming that all the megafaunal fruit species included in our analyses lost *all* their dispersers with the megafauna extinction. It is clear that functional dispersal for many of these species operates in present-day neotropical communities by means of diplochorous and alternative seed dispersal systems involving other agents such as scatter-hoarding rodents, tapirs, some primates and even bats [33]–[35]. However, the loss of seed dispersal by extremely large mammals may imply marked shifts in the patterns and consequences of seed dispersal for these plant species. The point is to what extent the ecology of megafaunal fruits can be understood without considering the relatively recent extinction of their primary dispersers and the dramatic changes in their life-history unfolded by this loss of mutualists. Therefore, we recognize that many of these plants actually have some legitimate seed dispersers, but we are interested in changes related to the extinction of their larger seed dispersers.

Certainly, the post-Pleistocene defaunation of neotropical megafauna has been extreme. By the end of Pleistocene, the South American fauna had at least 7 genera of large mammals from distinct orders with body mass ≥1000 kg [36], yet no one is present now. However, the megafauna is still extant in Africa with 5 genera (*Ceratotherium*, *Diceros*, *Giraffa, Hippopotamus,* and *Loxodonta)* and in Asia with 2 genera (*Elephas* and *Rhinoceros*). There is strong evidence that the extinct megafauna from the Pleistocene in South America included fruits in their diet or had mixed diets characteristic of browser species, presumably with a large fruit component [37]–[40]. This is a dietary pattern very similar to extant elephants, as revealed by isotopic analysis of enamel and bone remains [41]. Animal-dispersed fruits have been postulated to be bigger in the Paleotropics because their frugivore fauna is bigger than the Neotropical [42], but this implicitly ignores the fact that the extinct megafauna in South America was at least as diverse as the Paleotropical until the end of the Pleistocene [38], [43]. Thus, a proper comparison and characterization of fruit species in these areas should include megafauna-related taxa.

In this paper we address the megafaunal syndrome hypothesis by giving an explicit definition and quantification of fruit traits of putative megafauna species from Brazilian plant communities, comparing them to extant and related species in other habitats and examining the ecological correlates of the syndrome. Our goal is to create a new and operational concept of the megafaunal syndrome, collect evidence for phylogenetic and ecological patterns associated with megafaunal fruits, and hypothesize the potential consequences for the biology of the set of species involved in this peculiar type of interaction. We aim at formulating testable predictions about the potential effects of the loss of megafauna dispersers assuming that they were important to seed dispersal. Our predictions are based on (1) a rigorous characterization of fruits that may have depended extensively on large extinct mammals for much of their dispersal and (2) morphological and ecological correlates across fruit species from different plant families that can be easily interpreted in the context of the megafauna syndrome hypothesis. The specific questions we address are: 1) does the megafauna fruit syndrome exist as a separate entity in natural communities? 2) what are the life-history and ecological correlates of survival of megafauna plants in present-day habitats? 3) which potential genetic and ecological consequences can be predicted in the absence of the megafauna dispersers and, finally 4) how did plants survive the extinction of their main seed dispersers?

### Definitions

In the subsequent sections we use the following operational definitions and terms.

#### Anachronisms

These are extant interactions between animal frugivores and plants involving traits that show striking unfit patterns to an extant fauna. Anachronisms are different from present-day dispersal systems that work on exapted traits [6], [44]. We emphasize the difference because exapted interactions typically have functional effects on plant fitness despite having evolved out of this functional context. In anachronic seed dispersal systems, the functional role of fruit traits on present-day interactions with frugivores is probably marginal, being replaced in part by abiotic factors (wind, gravity, water, runoff, etc.) and determining secondary seed dispersal [19], [34]. Secondary seed dispersal by small- and medium-sized scatter-hoarding rodents might have been fundamental for the persistence of megafaunal fruit species after extinction of their primary seed dispersers [32, and references therein]. Furthermore, interaction with humans has been central to the extensive maintenance of these species over relatively large geographic areas, a fact not explored in previous discussions of anachronic dispersal systems. In all these cases, profound changes in seed dispersal patterns are likely to have occurred.

#### Megafauna

These are faunistic elements (taxa) of the frugivore communities interacting with a given plant species that characteristically have a large (>1000 kg) body mass [36], [45]–[46]. We are using here this restricted definition from Owen-Smith [45], [46] rather than the more broad advanced by Martin and Klein [36] (>44 kg) because of its biological basis. In South America, megafauna include primarily the large terrestrial mammals (proboscideans, extinct xenarthrans, and extinct orders such as Notoungulata)[47]. This immense diversity of large megafauna was driven extinct by human hunting and climate change in the last ice age [48]–[51].

#### Megafaunal fruits

In order to compare megafaunal fruit characteristics with other fruits we need unambiguous criteria to characterize the syndrome. We used the criteria that define African elephant fruits [12], [27], [52]–[58; also see 25,59] and searched the literature and our own data for Brazilian species that fit this criteria. These species are hereafter defined as megafaunal fruit species for subsequent analysis. Elephants can be considered a useful conceptual model for frugivorous megafauna due to their size, ecomorphology, generalized diet, as well as the quality of the information regarding their dietary habits. Indeed, paleontological evidence based on isotopic analysis indicates extremely similar dietary composition for, e.g., gomphotheres and elephants [41]. We defined megafaunal fruits as two fruit types [27]; Type I includes fleshy fruits 4–10 cm in diameter with up to 5 large seeds (generally >2.0 cm diameter), and Type II includes fleshy fruits >10 cm diameter with numerous (>100) small seeds. It is important to note that this definition does not assure that megafaunal fruits will be the larger fruits in a given community or clade. For example, some palms and Lecythidaceae species produce very large fruits without fleshy pulp [33], [60] and therefore they are not megafaunal fruits, but typical rodent-dispersed, nutlike fruits. In addition, by using fruit traits related to consumption by Paleotropical extant megafauna, these criteria are external to the species sampled so that they can be applied without circularity. This departs from Janzen and Martin [7] original definition, which is too vague because it includes a broad range of fruits which actually have reliable, present-day, main dispersers [17], [18]. Thus, our definition restricts the analysis to megafauna-dependent species as described by Barlow [8], [17], who acknowledges this broad gradient of reliance on megafauna dispersers among higher plants' fruits. Barlow [8] has termed these fruits ‘overbuilt’. However, most likely, the extinct megafauna included a broad range of fruit types in the diet, with species also eaten by other smaller frugivores such as scatter-hoarding rodents, primates, bats, and birds. We focus here on megafauna-dependent fruit species, and acknowledge that a gradient of reliance on megafauna for dispersal can probably be found among these fruit species (moderate, substantial and extreme anachronisms, *sensu* [8], [17]). For these fruit species, the absence of their main seed dispersers from the frugivore community might represent dramatic consequences in terms of restricted dispersal, disproportionate mortality of fruits and seeds due to pathogen attack, or severely altered seed shadows in terms of limited dispersal distance or increased aggregation of the seed rain. Our narrowed definition is not only consistent with reports of elephant-dependent species [58], [59], [61], but also with other present-day megafauna dispersers [4], [25], [29], [58], [62]–[65]. Therefore, megafaunal fruits are “outlier” fruit species in extant plant/frugivore communities [8], [17]. They are outliers because of functional lack of fit to characteristic present-day dispersal syndromes (suites of fruit traits associated with major dispersal by a particular group of vertebrate frugivores in the community). Here, we explore the morphological-basis for this functional lack of fit. However, we emphasize that functional lack of fit might be caused by differences in fruit structure, design, size or display, phenology, life form, microhabitat occupancy, biogeographic provenance, or any other trait that makes the species not particularly associated to a given extant frugivore species or group of species.

## Results

### Characteristics of megafaunal fruits

**Figure 1:**
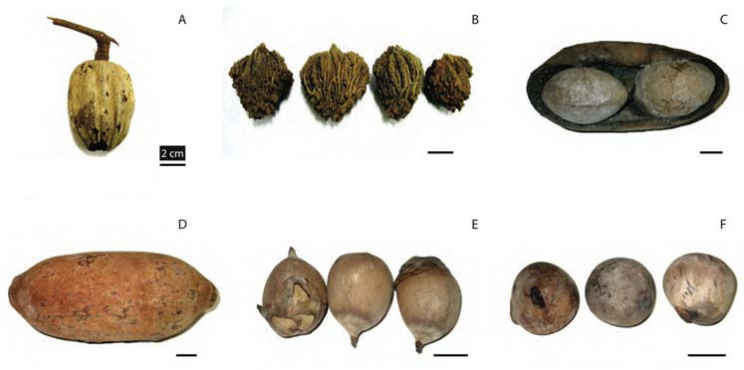
Examples of megafauna fruits and seeds. a, *Lacunaria jemmani*, Quiinaceae. b, *Parinari montana*, Chrysobalanaceae (seeds); c, *Caryocar villosum*, Caryocaraceae, fruit split open with two seeds; d, *Theobroma grandiflora*, Malvaceae; e, *Attalea martiana*, Arecaceae; f, *Phytelephas macrocarpa*, Arecaceae (seeds). Black line is 2 cm length. Photos from specimens at Herbarium João Murça Pires (MG) of the Museu Paraense Emílio Goeldi, Belém, Brazil; by PJ.

**Figure 2:**
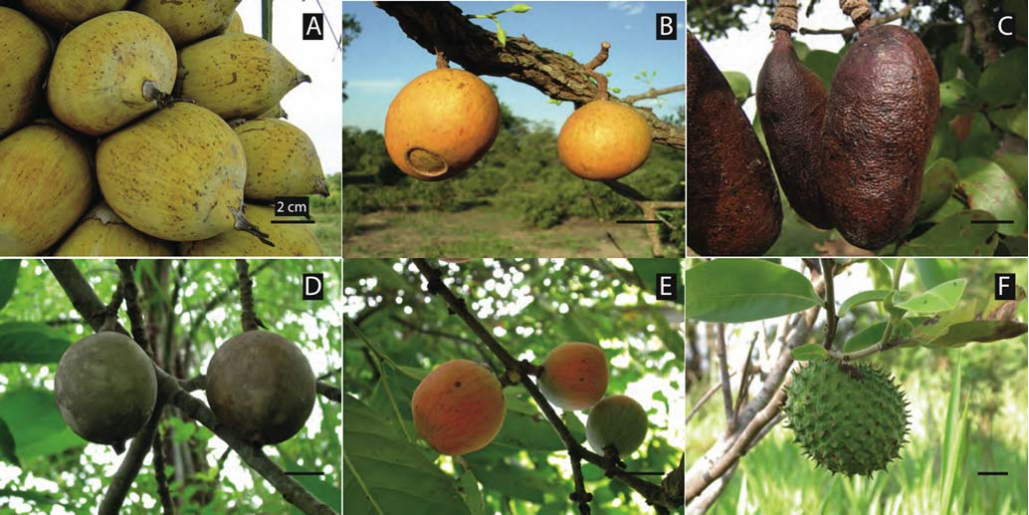
Fleshy fruited megafaunal-dependent species illustrating size, shape, and color variation. a, *Attalea speciosa*, Arecaceae; b, *Mouriri elliptica*, Melastomataceae; c, *Hymenaea stigonocarpa*, Fabaceae; d, *Genipa americana*, Rubiaceae; e, *Salacia elliptica*, Celastraceae; f, *Annona dioica*, Annonaceae. Black reference line is 2 cm length. Photos from Fazenda Rio Negro, Pantanal, Brazil; by PJ, MG, and Camila I. Donatti.

We identified 103 megafaunal fruit species (Table 1) fitting our criteria of Type I or Type II fruits out of 1361 sampled species (see Methods). Our definition allows the inclusion of extremely large fruits with many small seeds. However, even some of the multi-seeded megafaunal fruits have relatively large seeds (e.g., *Hymenaea*, *Theobroma*, with >5 seeds/fruit, and individual seeds >10 g mass) (Fig. 1 and 2).

**Table 1:** Relative representation of the megafauna syndrome fruits in angiosperm families and summary of fruit trait variation among species.

Order	Family	Megafauna species	Example species	LENG (mm)	DIAM (mm)	FRFM (g)	SEEDS	SEEDM (g)
Arecales	Arecaceae	22 (51.2)	*Attalea phalerata*	59.1±5.1	36.9±1.8	35.2±5.2	1.3±0.1	18.3±3.3
Poales	Bromeliaceae	2 (100)	*Ananas comosus*	205.0	…	1475.0	1000.0	…
Celastrales	Celastraceae	1 (100)	*Salacia crassifolia*	44.0	31.5	…	2.0	1.8
	Icacinaceae	2 (28.6)	*Poraqueiba paraensis*	72.5	43.8	110.0	1.0	13.7
Ericales	Sapotaceae	11 (40.7)	*Pouteria caimito*	73.6±6.9	54.2±8.3	410±210	1.9±0.3	10.0±4.3
	Lecythidaceae	1 (50.0)	*Couroupita guianensis*	210.0	200.0	…	…	0.28
Fabales	Fabaceae	24 (100)	*Hymenaea courbaril*	163.2±36	29.3±2.7	125.9±57.7	11.4±0.3	7.8±3.3
Gentianales	Apocynaceae	1 (7.7)	*Ambelania acida*	150.0	151.0	152.0	155.0	0.05
	Rubiaceae	1 (2.1)	*Genipa americana*	115.0	75.0	350.0	200.0	0.07
Magnoliales	Annonaceae	7 (24.1)	*Annona crassiflora*	130±7.9	107.9±10.7	1291.7±372.6	130.0±30.9	0.5±0.1
Malpighiales	Humiriaceae	2 (100)	*Endopleura uchi*	65.0	52.5	60.0	2.0	…
	Chrysobalanaceae	4 (36.4)	*Licania tomentosa*	94.4±12	75±25.6	…	1.0±0.0	76.0±64.1
	Caryocaraceae	3 (100)	*Caryocar brasiliensis*	81.7±9.3	75±8.7	…	2.5±0	13.1±9.2
	Clusiaceae	3 (27.3)	*Platonia insignis*	78.3±15.9	66.7±16.7	195.0±127.5	…	11.2±7.0
	Flacourtiaceae	1 (10.0)	*Carpotroche brasiliensis*	130.0	110.0	…	100.0	0.66
	Quiinaceae	2 (100)	*Lacunaria grandiflora*	110.0	71.5	…	…	…
Malvales	Malvaceae	6 (42.5)	*Quararibea cordata*	120.0	121.0	122.0	125.0	…
			*Theobroma grandiflorum*	174±35.3	93.2±15.9	975±454.8	34.4±14.7	12.1±11.9
Myrtales	Myrtaceae	4 (15.4)	*Eugenia cambucarana*	58.2±10.6	57.6±11.9	…	5.8±2.3	…
Sapindales	Anacardiaceae	4 (21.1)	*Anacardium occidentale*	61.3±10.7	36.4±3.9	47.5±8.8	1.0±0.0	3.1±0.7
Solanales	Solanaceae	2 (6.7)	*Duckeodendron cestroides*	86.5	61.3	…	350.5	6.7

Megafauna species include total megafaunal species in our dataset (outside parentheses) and the proportion of megafaunal species for a given order in our dataset (inside parenthesis). For all fruit traits, the value provided is the average±SD for megafaunal fruits in our dataset. LENG = length, DIAM = diameter, FRFM = fruit fresh mass, SEEDS = number of seeds and SEEDM = seed mass.

These are the 20 families, out of 93 families with data available, including species with both megafauna and non-megafauna fruits. Figures are mean±1 SE (*N*) of fruit traits of megafauna species; SE values were omitted for families with <3 species but means are reported otherwise. Classification follows [122] with modifications by [123].

**Figure 3:**
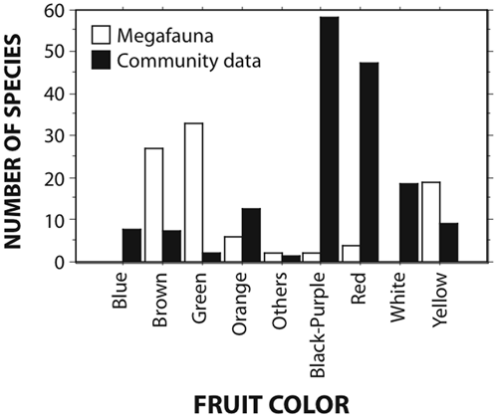
Frequency of megafauna species with different fruit colors (blank bars) compared to the summed frequency in different communities (filled bars). The available data for Manu (Peru), Monteverde (Costa Rica), Florida, Europe [118] New Zealand [124], and Brazilian Myrtaceae [125] have been pooled to characterize the color distribution pattern in extant communities.

Most megafaunal fruits with available data on characteristics (Table 1) are drupes or drupaceous (40.1% of the species), berry-like (29.9%) or legumes (18.6%). Contrary to fruit assemblages from different communities, the range of fruit colors of megafaunal species is very restricted, predominantly brown, brown-red or brown-greenish (24.8%), green, green-gray (34.5%) or green-yellow (12.9%) or different tones of yellow or yellow-green (21.5%) (Fig. 3; see Figs. 1 and 2). This contrasts markedly (χ^2^ = 408.78, *P*<0.0001) with the distribution of fruit color frequency in different communities worldwide, which are predominantly black-purple or red (Fig. 3), except for New Zealand communities where blue and white colors are very common. The restricted color pattern holds when comparing local sites in south and southeastern Brazil; the combined relative frequency of orange, brown and green colors in a lowland Atlantic forest site (Intervales Park) is 23% (*N* = 174 species), contrasting with 46% (*N* = 54) for Pantanal (Rio Negro), where megafaunal fruits are much more frequent. The relative frequencies of red-colored fruits are 24% and 5%, respectively. Other colors (e.g., yellow, black, and bicolored fruits) are represented in similar proportions. The differences in relative frequencies of the seven colors are highly significant (χ^2^ = 14.16, *P*<0.003, d.f. = 6).

**Figure 4:**
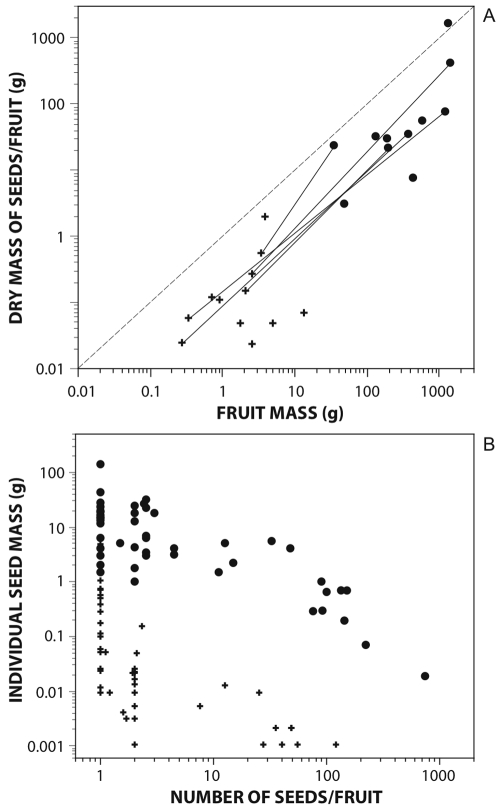
Bivariate plots of fleshy fruit traits for megafauna and non-megafauna species. Dots, megafauna-fruit species; +, non-megafauna fruited species. (A) dry mass of seeds per fruit and fruit mass. Intrafamilial comparisons are indicated by connecting lines between dots and +s; (B) individual seed mass and number of seeds per fruits.

Megafaunal fruits are characteristically heavy (Table 1), varying in form between spheroid drupaceous designs and elongate legume-like forms up to 50–1000 g total fruit mass. This results in very high seed loads/fruit, with total seed(s) mass/fruit increasing with fruit mass (Fig. 4a) (*R* ^2^ = 0.9221, *F* = 65.12, *P*<0.0001, d.f. = 2, 11); a trend also patent when comparing intra-familial contrasts (Fig. 4a). The slope of the relationship between seed load/fruit and fruit mass (Fig. 4a) does not depart significantly from a 1∶1 trend, suggesting seed load is an isometric function of fruit mass for these species. In addition, they typically show a larger seed load/fruit relative to non-megafaunal species. The mass of seeds/fruit ranges for megafaunal species between 0.2%–97.4% of the total fresh fruit mass, while the comparable range for non-megafaunal species is 0.1%–8.9%. However, this is the simple result of increasing total fruit mass, not increasing the relative seed load/fruit (Fig. 4a); thus, there are no differences between megafaunal and non-megafaunal species in seed(s) mass/fruit when accounting for variation in fruit mass (*F* = 2.11, *P* = 0.17, d.f. = 2, 11 for the *a posteriori* contrast with fruit mass as the covariate).

There is also a similar trend in fruit design between megafaunal and non-megafaunal species when comparing the allocation of seed number/fruit and individual seed mass. As expected, a negative trend between both variables is evident in the two groups (Fig. 4b), with individual seed mass decreasing linearly with increasing fruit seediness (*F* = 126.0, *P*<0.0001, d.f. = 3, 87). Yet megafaunal species have significantly larger seeds when controlling for variation in seediness (*F* = 8.36, *P* = 0.0048, d.f. = 1, 89 for the difference in slope between megafaunal and non-megafaunal species, Fig. 4b).

### Ecological and life-history correlates of the megafaunal anachronism in seed dispersal

**Figure 5:**
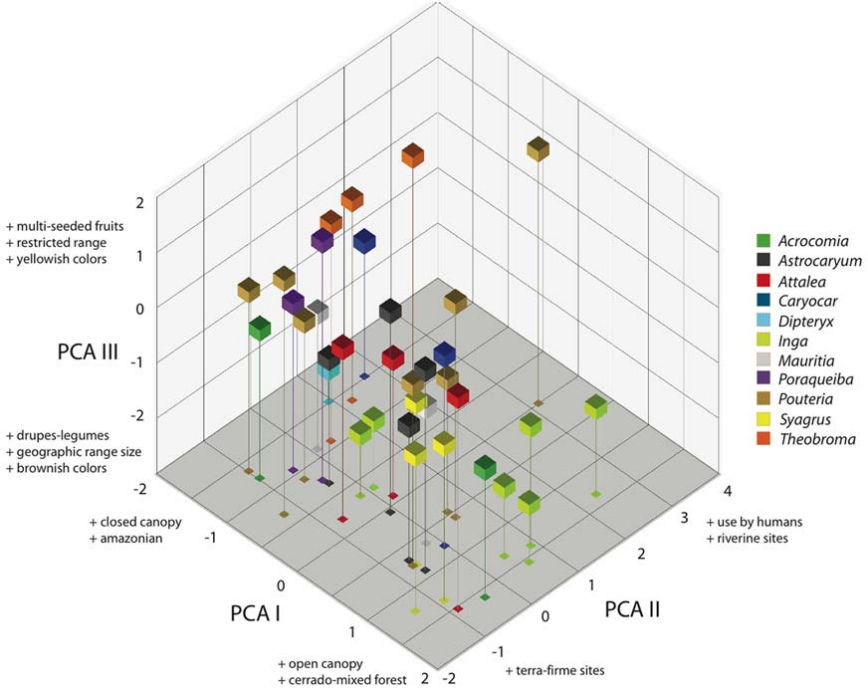
Principal components analysis of ecological and life-history variables of megafauna fruit species. Only genera (*N* = 11) with several species available for the analyses have been included. Cubes indicate the relative positions of individual species on the space defined by the three first principal components. Axes are labeled with short descriptions of the variables having larger loads (>0.40) on them.

Megafaunal species span a wide range of ecological and life-history traits. An ordination of their ecological and fruit traits (Fig. 5) revealed characteristic associations closely related to the taxonomic relatedness. Congeneric species clustered together in the ordination. The PCA with the first three significant components accounted for 78.7% of total variance. The first component was associated to fruit type and usage by humans, with increased human use related to multi-seeded fruits with greater relative amount of pulp/fruit (e.g., *Theobroma* spp.). A large group of species chiefly with drupaceous and/or legume-like fruits clustered on the positive side (Fig. 5). PCA II was associated to habitat distribution and geographic range, species with extensive geographic areas and inhabiting cerrado or mixed forest vegetation having positive loads on it (e.g., *Inga* spp., *Syagrus* spp.). Species with Amazonian distribution, associated to closed canopy forest (e.g., some *Astrocaryum*, *Acrocomia*, *Dipteryx*, *Pouteria* and *Poraqueiba*) had negative scores on this component. PCA III was associated with fruit color and habitat type; species with multi-seeded fruits, chiefly legumes, and dull-colored, brownish pulp had positive loads on it; species with bright fruit color, greenish-yellowish, and associated with *terra-firme* forest (e.g., some *Syagrus*), had negative loads on it.

Associations among ecological variables and fruit traits across species were tested by randomization (Table 2). Use by humans was significantly correlated with fruit mass and seediness. Geographic range was also positively correlated with seediness and negatively correlated with seed length (Table 2). All the remaining correlates were not significant. Most, if not all, the megafaunal fruit species share a level of human use, ranging from sporadic usage to extensive cultivation. The trends shown in Table 2 were consistent when examining within-family contrasts for these variables, although the small sample size limits the analysis. We therefore consider these trends with caution.

**Table 2:** Correlations between ecological variables (geographic range and human usage) and fruit traits of megafauna species.

Trait	Ecological variables	
	Geographic range	Human usage
Fruit length	0.2029^NS^	0.1213^NS^
Fruit diameter	0.0742^NS^	0.3828^NS^
Fresh fruit mass	0.0074^NS^	0.5757**
Number of seeds per fruit	0.4632***	0.3123*
Seed mass	−0.1774 NS	−0.1627 NS
Seed length	−0.2975 *	−0.0748 NS
Seed diameter	−0.1600 NS	−0.0451 NS

Correlations were tested with randomization tests (*N* = 10000 iterations). With Bonferroni correction:

*** , P<0.001;

** , P<0.01;

* , P<0.05;

NS , non-significant.

*N* varies for each trait due to missing values, with actual range 37–92 species.

### The taxonomic and ecological distribution of megafaunal fruits

We analyzed the data available for the *N* = 103 species characterized as megafaunal species (Supplementary Table S1) by any of the external criteria of [27] applied to fruit morphology. Megafaunal fruits appear repeatedly as subsets of species distributed among diverse angiosperm subclades. Megafaunal species represent a variable fraction of the species examined (*N = *1361 species sampled, including our megafaunal fruit dataset, the FRUBASE dataset and M. Galetti unpublished data) for different orders: Fabales (100%), Arecales (51.2%), and Ericales (36.4%); between 10–30% of species show megafaunal characteristics in Malvales (22.2%), Magnoliales (17.1%) and Celastrales (10.7%). Less than 10% of megafaunal species were recorded for Myrtales (9.5%), Solanales (6.7%), Gentianales (8.4%), Malpighiales (5.9%), Sapindales (4.8%), Rosales (4.6%), and Laurales (1.6%). This distribution indicates a widespread representation of megafaunal attributes in these taxa. Families with a high proportion of megafaunal species (Table 1) include Arecaceae, Sapotaceae, Fabaceae, Lecythidaceae, Humiriaceae, Caryocaraceae, some Malvaceae (i.e., formerly Bombacaceae and Sterculiaceae) and Quiinaceae. Among these families, the main genera with anachronic species are *Caryocar* (Caryocaraceae), *Attalea*, *Astrocaryum* and *Syagrus* (Arecaceae), *Andira*, *Dipteryx*, and *Hymenaea* (Fabaceae), *Pouteria* (Sapotaceae), and *Theobroma* (Malvaceae).

The frequency of megafaunal fruits is not constant across two distinct Brazilian ecological communities. In a single locality of lowland Atlantic rainforest (Intervales Park) [66], only 13% of the fleshy-fruited tree species (*N* = 246) have megafaunal characteristics (e.g., *Pouteria*, *Painari*, *Astrocaryum*), while in a Pantanal site, Fazenda Rio Negro, the proportion of megafaunal species reaches 30% (*N* = 147 species) [35].

In relation to ecological characteristics of the species in our megafaunal fruits dataset, 37.5% are from the Amazonian forest, 13.5% from Atlantic forest, 9.7% from *caatinga* and *cerrado* vegetation types, and 28.9% from semideciduous and mixed forest types (including a variety of formations). The main habitat types represented in our dataset are *terra firme* forest (54.8%) and riverine and swamp forest (16.3%). Most species are restricted to a small region (73.5%) and very few species show a continental range distribution (14.3%). Most species are trees (83.3%), frequently showing vegetative propagation or vigorous resprouting (84.2%).

### Comparisons between megafaunal fruits and other dispersal syndromes

**Figure 6:**
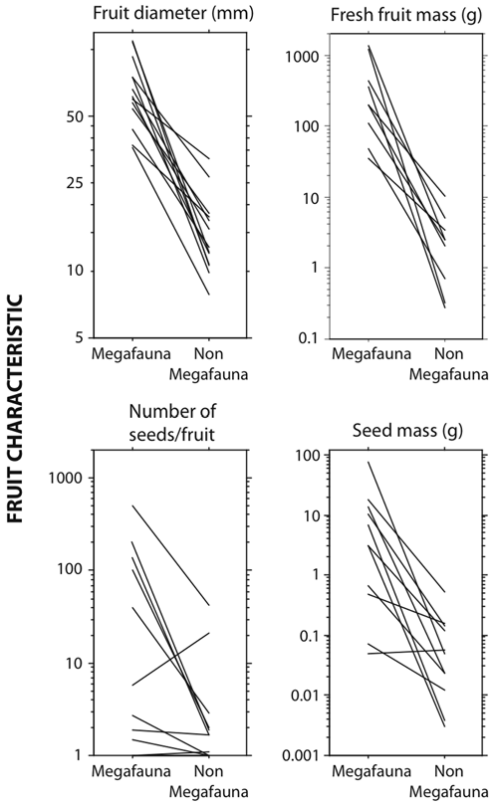
Within-family contrasts for fruit traits of megafauna and non-megafauna plant species. The pattern for fruit length was very similar to fruit diameter and has been omitted for clarity. Each line corresponds to the contrast (difference in mean trait value) between species of the same family with each syndrome.

To account for patterns of phylogenetic relatedness that might bias across-species comparisons, we contrasted the series of fruit phenotypic traits between megafaunal and non-megafaunal species by means of within-family and within-genus contrasts. Paired within-family contrasts between the two groups of species for the main fruit traits (Fig. 6) indicate consistent trends for larger fruit size in megafaunal species which is independent of family affiliation. This trend is very marked for fruit diameter and fruit mass and less so for individual seed mass; for all the four traits examined (Fig. 6) with data available, there is a significant trend for megafaunal fruits to have larger seeds and greater seediness, independently of the general trend for larger fruits (Table 3).

**Table 3:** Summary of the within-family contrasts of fruit traits between megafauna and non-megafauna species.

Trait	Contrasts		Binomial *P*
	+	−	
Fruit length	13	0	0.0001 ***
Fruit diameter	13	0	0.0001 ***
Fresh fruit mass	10	0	0.0001 ***
Number of seeds per fruit	11	2	0.0225 *
Seed mass	9	0	0.0001 ***

With sequential Bonferroni correction:

*** , P<0.001;

** , P<0.01;

* , P<0.05.

Positive contrasts for a trait indicate larger mean trait value for the megafauna species group within each family. The binomial test gives the probability of obtaining the observed proportion of positive contrasts. *N* = 24 families with both megafauna and non-megafauna species; *N* varies for each trait due to missing values.

The same trend can be confirmed for within-genus comparisons by contrasting congeneric species with megafaunal and non-megafaunal fruits. Larger fruits among megafaunal congeners are encountered in *Spondias* (Anacardiaceae), *Couepia* and *Licania* (Chrysobalanaceae), *Garcinia* (Clusiaceae), *Andira* (Fabaceae), *Eugenia* (Myrtaceae), *Pouteria* (Sapotaceae), and *Solanum* (Solanaceae). Among the palms, fruits are consistently larger for the megafaunal species of *Acrocomia*, *Astrocaryum*, and *Syagrus.* This is not the case for *Attalea*, with *A. dahlgreniana* and *A. dubia* having smaller or similar-sized fruits to *A. butyracea*, *A. funifera*, *A. oleifera* or *A. pindobassu*, which are dispersed by scatter-hoarding rodents. The rodent-dispersed *Attalea* species have extremely hard fruits with woody pulp. Among the *Eugenia* species, the megafaunal species (*E. cambucarana*, *E. klotzchiana*, *E. neoverrucosa*, *E. stipitata*) have fruits larger than other congeners having mixed disperser coteries with seed-caching rodents and primate frugivores.

We have less information for within-genus contrasts in seed mass, but both *Astrocaryum* and *Syagrus* non-megafaunal species have seeds <10 g, contrasting to megafaunal species, with seeds >15 g. Similar trends are observed in *Licania* (<2.0 g and >10 g, respectively). The trend is especially evident for drupaceous fruits (e.g., palms), although data are not available to test for differences in seed mass for berry-like, multi-seeded fruits (e.g., with >10 seeds/fruit). We should expect these species not to differ in individual seed mass, only in total fruit size, seediness and, consequently, total seed load/fruit (see e.g., Fig. 4b).

## Discussion

The megafauna syndrome hypothesis can potentially provide a broadened framework to analyze seed dispersal syndromes, resulting in an intellectually richer scenario by advocating an historical component for present-day interactions. Our analysis revisits and refines the megafaunal seed dispersal syndrome after Janzen and Martin [7; also see 8], [Bibr pone.0001745-Barlow1] [17], aiming at building an operative definition and provide, based on this definition, a start for the understanding of ecological benefits of seed dispersal by large mammals and the consequences of megafauna extinction for large-fruited plants.

We distinguished a few fruit attributes that can be used to determine if a species can be considered a fruit dispersed by the extinct megafauna. Being based on the current interactions of extant megafauna herbivores and fleshy fruits [27], [32], [52], [56], [58], [67], our approach provides a rigorous framework to analyze the “unfit” fruit traits in some neotropical taxa. We identified two distinct lines of fruit-trait variation that might have represented increased dispersal advantages over non-megafauna related taxa: production of large fruits packaging extremely large individual seeds (e.g., *Annona*, *Theobroma, Parinari, Caryocar*) and production of extremely large fruits packaging large numbers of moderate-sized seeds.

Our analysis suggests that the megafaunal syndrome is extensively represented in a few higher taxa (e.g., Fabaceae, some Malvaceae, Sapotaceae) but other families have a few species with megafaunal fruits closely related to species dispersed by present-day frugivores (e.g., Arecaceae, Myrtaceae, Anacardiaceae, Annonaceae). However, future research is needed to accurately estimate the frequency of megafaunal fruits in different higher taxa and the percentages in our sample should be considered with caution since they are not based on a systematic sampling of local floras. Our data for two Brazilian localities indicate that megafaunal fruits can be relatively common (e.g., up to 30% of species) in Pantanal plant formations but with a marked decrease in the Atlantic rainforest, where frugivorous birds are common seed dispersers [68].

### The advantages of seed dispersal by megafauna

Megafaunal fruit species represent a wide range of species that share a characteristic fruit design that cannot be readily interpreted in terms of ongoing ecological interactions with seed dispersers [8], simply because (1) their fruits are intensively harvested only by large mammals when they exist or (2) no extant vertebrate (except tapir and livestock) can act as seed disperser by endozoochory, due to fruit design limitations. By interacting with extremely large frugivores, these fruit species might have escaped the pervasive size constraints that may keep seed size below a certain threshold value so that it does not compromise dispersal ability. Size/dispersal ability tradeoffs have been repeatedly documented in plant fruits [69]–[71] and are certainly observed in megafaunal fruits but we found that megafaunal species can pack up to 85% seed load per g of fruit with up to 140 g seed/fruit. Only by relying on large frugivores free of size constraints can plants extensively disperse seeds larger than the 3.5–4.0 cm diameter limit apparently imposed by present-day Neotropical vertebrate frugivores [32], [41], [65], [67], [72]–[74]. This is a seed size limit similar to the 2.8 cm limit for ingestion by African forest ungulate cephalophines [54]. Very few extant Neotropical dispersers, like tapirs, can have larger seed loads per scat, allowing the dispersal of much larger individual seeds. Large seed size, in turn is a fundamental trait for plant species to survive in periodically flooded or dry areas, especially on nutrient-poor soils like those of *igapó* forest or cerrado [75]; these are areas with a high frequency of megafaunal-dispersed species. In this context, we show that the frequency of megafaunal fruits is higher in a flooded area (Pantanal) than in an Atlantic forest site. Future studies should investigate the regularity and the ecological bases of the variation in number of megafaunal-fruited species across ecological communities. While dispersal by megafauna might select for specific fruit traits, it is well established that seediness and seed mass are also subject to multiple ecological influences [32], [76]. Our analysis reveals consistent trends for phylogenetically-restricted comparisons of fruit mass, seed mass and seediness but we cannot discard these traits evolving in concert with other ecological characteristics.

Large extinct mammals with size not limiting the consumption of megafaunal fruits like those analyzed here include most of the terrestrial xenarthrans (*Glossotherium*- and *Lestodon*-like genera, megalonychids, and megatherids [77], large sized proboscideans (gomphotheres, mammoths, mastodons) [40], and other groups like camelids, litopterns, toxodons and equids [47]. Among the largest ground sloths, *Megatherium* and *Eremotherium*, the cranial traits coupled with the post-cranial ecomorphological design point to strongly frugivorous-browser diets related to high browsing habits, while probably *Eremotherium* was more able to handle softer food [38], [39], [78]. There is compelling evidence that the megafaunal fruit species interacted with extremely large terrestrial frugivores such as ground sloths [38], gomphotheres, mastodons, and mammoths [37], [39], [40] and smaller-sized but large semi-terrestrial atelines [79]. Evidence for diversified plant-based diets including relatively large fractions of fruit material as well as plant remains of fleshy-fruited shrubs and trees come from ecomorphological studies of fossil remains [38]; evidence from coprolite and isotopic analysis [20], [28], [29], [41], [80]; as well as from studies of present-day large Paleotropical seed dispersers (elephants, rhinos, cassowaries) [12], [25]–[29], [42], [54], [56]–[58], [63], [81]. This evidence points not only to sporadic frugivory among megafauna taxa, but also to an extended reliance on fruit food by these animals. Most of these species were larger than the largest present-day terrestrial megafauna, with the exception of the African elephant [32], and included at least 6 families with 13 genera in the Neotropics with body mass >1000 Kg [38], [39], [82].

A distinct characteristic of megafaunal fruits is that for a given number of seeds/fruit, the fruits pack significantly larger seeds than non-megafauna taxa. This particular fruit design, combined with large frugivore size, would imply the potential dispersal of large numbers of relatively large seeds. Thus, an average-sized terrestrial extinct megamammal could have dispersed thousands of large seeds of any species, probably scattering them over a sizable area, based on estimates available for elephants and rhinoceros [27], [54], [81] and extinct megafauna body sizes [38], [39], [82]. Nowadays, only tapirs, can have large seed loads per scat in the Neotropics [62], [67], [83]. Thus, megafaunal fruit species could take advantage of interacting with frugivores capable of dispersing seed loads much larger than those dispersed by extant frugivores and including much larger individual seeds, ultimately entailing increased advantages in terms of seedling vigor and survival prospects. Besides, large seed size also allows survival of partial consumption by seed predators [84]. Therefore, megafauna frugivore species were most likely reliable dispersers by providing the dissemination of large quantities of seeds over enormous areas, involving frequent events of long-distance dispersal.

Additional advantages of the ability to disperse extremely large individual seeds would be related to the possibility that these large mammals acted as long-distance dispersers of these large seeds. No present-day Neotropical frugivore, with the probable exception of tapirs [62], [85] and introduced species (e.g., feral pigs), is likely to provide dispersal services combining reliable consumption and removal of seeds >2.5 cm diameter and potential dispersal on a regular basis (i.e., not sporadic long-distance seed transport, as recorded by [86]) at scales >10^3^–10^4^ m away from the maternal plant. In fact, both the medium- and large-sized gravigrade species, such as ground sloths, were able to do long-distance dispersal [39]. Dispersal of large-seeded species can be accomplished by some present-day frugivores (e.g., large bats, toucans and large cracids, ateline monkeys and scatter-hoarding rodents) but most likely with much fewer seeds moved, often in short-distance events around 10^1^–10^3^m [32], [87]–[89].

### The survival of megafaunal fruits

The consequences of disperser extinction are just starting to being assessed in depth for some present-day plant-frugivore interactions [4], [61], [64], [65], [72], [90], [91]. The evidence points to three main types of potential bottlenecks that frugivore extinction might cause on plant population viability, and they illustrate analogous ways for pervasive consequences of the megafauna extinctions. First, we should expect a net decrease in the quantitative aspects of dispersal, i.e., a significant decrease in the total number of seeds successfully dispersed away from the maternal plant, especially for large-seeded species [65], [67], [72], [92]. Second, the loss of large frugivores may have a dramatic impact on plant demography by severely altering the seed shadow pattern, i.e., resulting in limitation of dispersal in both distance and area (e.g., [61]). Third, loss of large frugivores probably caused parallel effects on population genetic structure by restricting gene flow via seeds.

There is indeed evidence that the loss of large-bodied frugivores, capable of transporting large numbers of large seeds over long distances, has caused increased population differentiation because of a dramatic loss of potential for gene flow via seeds [93]. Recent molecular analysis of genetic variation and structure of species with megafaunal fruits tends to confirm this prediction [93] and several megafaunal fruits in Brazilian cerrado vegetation present a similar trend in genetic variability. For instance, *Eugenia dysenterica*, *Calophyllum brasiliense*, *Caryocar brasiliensis*, and *Vouacapoua americana* all present moderate levels of genetic variability within population but high genetic differentiation among populations, combined with presence of private alleles, reflecting limited gene flow via seeds [94]–[99].

How to survive 10,000 years without dispersers or with poor dispersal services? The mass extinction of megafauna frugivores in South America occurred approximately 10500 yr BP, with more recent extinction on islands [51]. This could involve de facto survival over 100–200 generations for some of the tropical species involved, which is certainly anomalous [18]. Although we cannot exclude that a few plant species have already gone extinct after the Pleistocene megafauna extinction, the persistence of many megafaunal species needs an explanation. Our data suggest most species relied on secondary dispersal or sporadic primary dispersal by generalist frugivores. While poor and limited dispersal by endozoochory can be observed in the field for a few species with megafaunal fruits (e.g., *Hymenaea courbaril, Duckeodendron cestroides* [100], [101]), it is relatively frequent to record dispersal by gravity, water, scatter-hoarding, or favored by human harvesting, in addition to vegetative propagation [19], [32], [67], [86], [87], [100]–[104]. These are diplochorous systems involving multiple and varied dispersal vectors [34]. For instance, most megafaunal fruit species from Pantanal formations are dispersed now by a combination of seasonal flooding and sporadic consumption by tapirs, cattle, or feral pigs [35]. This impairs their dispersal if we consider the action those extinct megafauna dispersers could have on these species: the removal of extremely large quantities of fruit and extensive dispersal in distance. No extant species in Neotropical communities has this potential effect of dispersal by endozoochory, despite being now functional in performing dispersal services for megafaunal fruit species.

In addition, interactions with humans (paleoindians and extant Indigenous populations, cf. [105]) have probably been central in the maintenance and dispersal of a fraction of megafauna species, especially those with multi-seeded fruits. The effects of interactions with humans were probably less pronounced for the large-fruited and large-seeded species, as suggested by the correlation analysis of ecological traits indicating a significant association of seediness, human usage, and geographic range. The long-term interactions of megafaunal fruits with humans (see e.g., [105]) might have influenced not only the local persistence of a number of species, but also their geographic range and population sizes. These patterns, however, would require additional evidence and tests with a larger number of species.

Finally, environmental influences in some habitat types (e.g., the Pantanal and *várzea* and *igapó* formations in Brazil) probably caused secondary seed dispersal by flooding, acting as a surrogate disperser for megafaunal species, and this can explain the high frequency of these species associated with flooded areas [17], [19]. The ability of species to successfully establish in flooded forest relies on dispersal of relatively large seeds able to develop tall seedlings in a short period of time [75] and megafauna frugivores were probably central in the successful recruitment of large-seeded species in these habitats. Recent demographic simulations [35] suggest that the above factors, resulting in limited and marginal dispersal, might allow long-term local persistence of megafauna-dependent species.

For the smaller-sized fruit species (e.g., Sapotaceae, Anacardiaceae, some Arecaceae), mammals are the main current frugivores legitimately dispersing the seeds, and only a few species have mixed disperser assemblages involving birds and mammals (see [32]). For these, the impact of present-day extinction of the medium-sized mammals and large frugivorous birds can be as dramatic as the megafauna extinction [16], and we still have a very limited understanding of its effects [14], [15]. Most likely, megafauna species with multi-seeded fruits and small seed size have escaped the pervasive effects of selective extinction of the large megafauna by a combination of reliance on smaller-sized frugivores able to handle the seeds, human-mediated dispersal, vigorous vegetative sprouting, and increased importance of secondary dispersal by runoff and flooding. Moreover, some species also are so well-protected against seed predation beneath parent plants that distance-limited dispersal in present-day scenarios does not determine post-dispersal seed mortality (e.g., large-seeded *Attalea speciosa* [35] ). Our results indicate that megafauna species include a highly heterogeneous assortment of fruit morphologies and ecological characteristics and so we have to consider a diverse array of potential responses to extinction of their major dispersers. Whether the extinction of major, presumably efficient, dispersers led to serious disruption of the plants populations is probably related to the degree of reliance on megafauna dispersal [17], so that a wide gradient of megafauna-dependence patterns can be envisaged. Major effects would be expected in extreme megafauna-dependent species.

### Concluding remarks

One of the pervasive consequences of extinction of the major seed dispersers of a plant would be a collapse in the natural regeneration cycle, a severe bottleneck in one of its sequential stages of recruitment, and a shortening of the seed dispersal distances leading to loss of genetic variation. The large post-Pleistocene mass extinction of a diverse megafauna [48], [106], whether caused by humans or not, presumably had a dramatic imprint in plant populations in the form of major changes in their demography, recruitment patterns, and regional distribution. Certain aspects of the reproductive behavior of megafaunal fruit species have been extremely relevant to assure their survival to the extinction of their major seed dispersers. Many species of megafaunal fruit show vigorous resprouting and vegetative growth following trampling or clear-cutting and this character has certainly favored persistence despite the extirpation of megafauna frugivores [4], [35]. In addition, suboptimal dispersal, whether sporadic or more regular by abiotic factors [18], [19], most likely contributed to a minimum recruitment necessary for population persistence, as suggested by recent numerical simulations [35]. Most of the megafauna fleshy-fruited species considered here rely on present-day small- or medium-sized mammals such as large primates, tapirs, and introduced feral pigs and livestock for successful regeneration; many are scatter-hoarded by large rodents [87], [100]. In this situation, the fast-paced extirpation of these large-vertebrate groups in present-day forest remnants poses a serious threat for the preservation of the peculiar elements of the flora represented by megafauna-dependent plant species [4], [21], [72]. In addition, our data reveal an important role of humans in the maintenance and dispersal of a subset of the megafaunal species, particularly the large-fruited, multi-seeded taxa; these fruits have been probably more amenable to human use by yielding larger pulp loads/fruit relative to their drupaceous counterparts. Anachronistic interactions are an important component of present-day plant-frugivore communities, yet we know very little of how they shaped fruit traits and regeneration strategies of the participant species. Understanding the functioning of megafaunal fruit species in present-day communities can be advanced in the future with the help of comparative analyses of different communities with and without native megafauna, theoretical models of dispersal dynamics, and analysis of population genetic variability and spatial patterns. Since many areas worldwide are facing fast-paced defaunation [16] it is imperative to understand the implications of past extinctions on the population structure of the living plants (see [15]) to predict the effects of ongoing extinction of the seed dispersers.

## Materials and Methods

Data on fruit traits were compiled from the literature and by direct sampling in the field. The area for field samples was located in different major Brazilian vegetation types in Pantanal (wetland with dry and gallery forests and cerrado), Caatinga (semi-arid, thorn savanna), Cerrado (savanna-like vegetation) and semideciduous forest and Atlantic rain forest. To assign a species to the megafauna group we compared if the traits fitted any of Feer's [27] typologies (Type I and II) for elephant fruits, as this provides an “external” criteria to evaluate a proper assignment. In total 103 species from 22 families and 46 genera spanning all Brazilian biomes were sampled (Supplementary Table S1). The dataset is based on references [107]–[116].

For species included in our survey, data are available for fruit length (LENG), fruit width (cross diameter; DIAM), fresh fruit mass (FRFM), number of seeds per fruit (SEEDS), and individual seed mass (SEEDM). To assess consistent patterns in fruit morphological trends for megafaunal fruits we compared these characteristics with confamilial or congeneric species in the large FRUBASE dataset [6] of fleshy-fruit traits of angiosperm species, including information for 910 species, as well as other non-megafaunal species not included in FRUBASE (75 species from Pantanal [35], and 356 from Atlantic rain forest; M. Galetti unpubl. data). FRUBASE is a long-term project maintained by one of us (PJ) and most of its information derives from literature sources on frugivory and seed dispersal. The megafaunal fruits dataset and the list of primary literature used for published data are available as Supporting Material (Supplementary Table S1), upon request from the authors, or from http://ebd10.ebd.csic.es/frubase/.

We first reported the frequency of megafaunal fruits among different taxonomic groups in our datasets by referring the number of megafauna-dependent species to the total species within each higher taxa in the reference dataset (the extended FRUBASE database). Here, we were not interested in providing accurate estimates of the frequency of megafaunal fruits in the Brazilian flora. Rather, our aim was to provide a coarse description of patterns of variation in the frequency of megafaunal fruits among higher taxa. We have investigated if (1) megafaunal fruits are restricted to a few taxa or widespread across many families and orders and (2) megafaunal fruits are more common in some taxa than others. When the literature source reported the range of a given variable we estimated the midpoint of the range and used it in subsequent analyses. Ecological and life-history information (Supplementary Table S1) was also compiled from literature sources [107]–[116] and from unpublished material (P. Guimarães Jr., M. Galetti, and P. Jordano, unpubl. data). Disperser types were categorized into broad classes: 1) birds, with plant species dispersed predominantly by avian frugivores; 2) mixed, including frugivorous birds and mammals in the disperser assemblage; 3) mammals, dispersed chiefly by mammalian frugivores (including those species dispersed by food-hoarding frugivores, mostly large terrestrial rodents) [117]. Thus, categories 1–3 define a gradient of increasing participation of mammal frugivores in the seed dispersal process of the plants (see [6]). Whenever possible we compiled data on life-history characteristics of the plants, including: 1) geographic range, coded in four ranks (restricted, 0–100×10^3^ km^2^, with distribution spanning 2–3 small Brazilian states; regional, 100×10^3^–1,5×10^6^ km^2^, spanning a Brazilian region; large, 1,5×10^6^–7×10^6^ km^2^, spanning 2–3 Brazilian regions; and continental, >7×10^6^ km^2^, extending over large areas of Brazil. 2) Usage by humans was coded in four broad categories: no use, if fruits are not consumed by humans; local harvesting, if consumption is recorded locally from wild trees in the neighborhood of human settlements; regional plantation, if cultivation of the plant is reported and it represents a frequent food item; and extended use, if the plant species has economic value. 3) Fruit type, was coded as drupe or drupaceous, berry-like, legume, other (including e.g., syconia). 4) Main vegetation type, coded as Amazonian rainforest, semideciduous forest, Cerrado vegetation, Caatinga, Atlantic forest, or mixed forest whenever the species is characteristic of several vegetation types. Fruit color was coded as in [118]. This information was largely compiled from literature sources [35], [107]–[116] and unpublished material (P.R. Guimarães Jr., M. Galetti, and P. Jordano, pers. obs.; C. Donatti and M.A. Pizo, pers. comm.).

### Statistical analyses

We used randomization tests [119], [120] to assess differences between megafaunal- and non-megafaunal species in fruit traits. We used *N* = 10000 resamplings and applied the Bonferroni correction when using simultaneous tests on several variables (i.e., testing for differences among disperser type categories for several fruit traits).

In addition to using the raw data for comparisons, we used within-family and within-genus contrasts for inferring differences between megafaunal and non-megafaunal groups without taking into account the patterns of phylogenetic relatedness. Due to the scarcity of data on megafaunal fruits and the irregular distribution of missing values we resorted to these binary contrasts to partially control the patterns of phylogenetic relatedness (see [6], [121] for a similar approach). We used a binomial test to assess significant trends in fruit length, fruit diameter, fresh fruit mass, number of seeds per fruit, and seed mass associated with megafaunal dispersal. We used 13 within-family contrasts to test if the proportion of positive contrasts (megafaunal fruits with larger values of the variable when compared to non-megafauna confamilial species) exceeded a random expectation of *P* = 0.50. For a reduced number of genera we used within-genus comparisons, but these were insufficient for a formal test. To test correlations among fruit morphology variables and ecological variables (geographic range size, and human usage) we used a randomization test (N = 10000 resamplings). We used a principal component analysis to obtain ordinations of fruit species according to morphological and ecological and life-history variables. The PCA was carried out on the transformed variables after standardization; we used library ade4 of the R package [120]. For the ecological and life-history variables we used those coded as meristic values (i.e., ordinal scale): fruit color, fruit type (coded from berry and beery-like fruits to legumes and drupaceous fruits), geographic range size, human use, vegetation type (ordered from Amazonian lowland rainforest to Atlantic Forest, mixed, and caatinga and cerrado vegetation) and habitat type (ordered from riparian to terra firme forest type). We omitted genera with only one species from this analysis, using *N* = 11 genera with two or more species.

Nomenclature and species names follow [122], with modifications from [123].

## Supporting Information

Table S1 Fruit characteristics of megafauna-dependent species.
(0.07 MB XLS)
